# Results of Lumbar Anesthesia in Gynecological Operations

**Published:** 1909-01

**Authors:** Frederich Brunner

**Affiliations:** Assistant at Professor Amann’s Gynecological Clinic at Munich


### Results of Lumbar Anesthesia in Gynecological Operations.

By FREDERICH BRUNNER,
Assistant at Professor Amann's Gynecological Clinic at Munich.

(Translated and reprinted from *Monatsscher. f. Geburts. u. Gyn.* Bd. XXIV, Heft 5).

SPINAL anesthesia has been for several years past carried out quite extensively, the reports of which are constantly being read. In January of this year, a review of this procedure from the surgical aspect appeared from Dr. Krecke. I will, therefore, that I may avoid repetition, be less exhaustive in my report on the result of lumbar anesthesia in gynecological operations at our clinic, which report I have made at the instigation of my chief, Professor Amann.

I may spare myself the subject of the technic of the puncture itself, for we always adhere strictly to the directions of Professor Bier. The height of the puncture was not always the same. In vaginal work, the site was chiefly between the third and fourth lumbar and also the fourth and fifth lumbar vertebra; while in laparotomies it was between the first and second or second and third. To anesthetise the site of the puncture itself seemed to us unnecessary, although advantageous in many cases to direct the ethyl chlorid spray on the site of the puncture, the white spot then formed also serving as a guide. After the injection, the patient is placed on her back with pelvis slightly elevated.

Almost immediately after injection the patient as a rule feels sensations of warmth, formication, numbness, and the like. These paresthesia symptoms tend to begin at the feet and extend upwards to the trunk. There soon arises a paralysis of painful sensibility to touch, heat and cold, while sensibility to touch, heat and cold, motility and reflexes is still preserved. The causes of the analgesia, according to Braun, are found in the action of the remedy upon the posterior nerve roots and portion of nerve trunks seated in the spinal canal. The influence of the anesthetic upon the sensory fibers is shown to be notably more intense than on the motor nerves, the latter being involved only in high degrees of local poisoning. The same may be said in respect to paralysis of the sensations of temperature and touch. The anesthetic mixes with the cerebrospinal fluid, spreads upward in the sac, becoming thereby more and more dilute, and paralyzes in succession the accessible root fibers until the dilution becomes too feeble to exert an anesthetic action.

A few words as to unpleasant collateral and after effects. Often we note anxiety, dyspnea, perspiration, trembling of limbs, convulsions, nausea and vomiting as collateral phenomena; while sequelæ include headache, backache, vomiting, insomnia, anorexia and weakness. Tuffier saw rise of temperature in the majority of cases. Death has even resulted. Collateral disturbances depend chiefly on rapid ascent of the injected fluid to the medulla oblongata. Subsequent phenomena are due doubtless to meningeal irritation.

After Bier's discovery, spinal anesthesia became widely employed, but was given up after a relatively short time. The latter was due to the toxicity of the cocain originally employed. The resumption of spinal anesthesia was due to the introduction of substitutes for cocain which were much less toxic. A great advance also made when suprarenal preparations were combined with the anesthetic; the contraction of the bloodvessels thereby causing the retention of the latter near the point of injection.

The unpleasant collateral and after effects have become much milder, or are absent altogether. The substitutes for cocain are eucain, tropacocain, alypin, stovain and novocain. We have used the latter especially. We employed spinal anesthesia in thirty minor and major gynecological operations, including curettage, emptying uterus after abortion, enucleation and thermocautery in inoperable carcinomata, amputation of pointed condylomata on the labia majora and minora, vagina and crural folds, extirpation of suppurating Bartholini's glands, carcinoma of clitoris and vulva, colporrhaphy, amputation of portis, posterior colpotomy in pelori exudate, vaginal total extirpation, the Alexander-Adams operation, ventrofixation, abdominal hernia, ovarian cystoma, myoma and implantation of ureters.

The duration of anesthesia was not over 50 to 60 minutes. The setting in of analgesia varied from 2 to 15 minutes. In four cases with correct technic, failure occurred in the following conditions: curettage of a uterus with marked terminal stenosis; in vaginal total extirpation (two cases), in which opening of the peritoneum and traction on the uterus were very painful, requiring inhalation of a little chloroform to complete the operation; and finally ventrofixation. The first patient was very nervous, had long been under treatment and had often been cureted under general narcosis. It should not be deemed a failure when the operation is of such a character as to outlast the anesthetic effects (over an hour); here it is necessary to add the effects of a little ether or chloroform.

Intervention in the perineum and ostium vaginæ have always been satisfactory; and the same holds good as a rule for intervention in the uterus itself, although curettage at the tube angles of the uterus was felt unpleasantly in two cases. Dilatation of the internal os, considered a painful procedure, was not felt. Krecke regards any intervention which necessitates traction on the peritoneum as almost always painful. We, however, performed the Alexander-Adams operation with opening of the peritoneal cavity and laparaotomy (both transverse and longitudinal incisions) with painless results. Legar and others would go no higher than the umbilicus, but we had two successful cases in which the incision reached above the navel (large myoma and ventral hernia). Peritoneal adhesions were detached and intestinal sutures placed, all without the slightest pain.

As to collateral symptoms: in the majority of patients no general disturbance occurred. One felt discomfort, another brief nausea, a third felt pressure on the head, and the like, but severe collapse or other notable phenomena were absent. As for after effects, these seemed to me more common and intense. Only eight patients presented nothing of the sort. As a rule, there was headache, more or less severe; vomiting often co-existed at the outset, which sometimes did not begin until the following day and even later. Two patients vomited daily for a week. In about ten cases pains in the loins were complained of, which sometimes did not begin until the following day and even later. Two patients vomited daily for a week. In about ten cases pains in the loins were complained of, reaching sometimes as high as the neck. Once a certain rigidity of the neck was noted.

Now for a criticism of medullary anesthesia. It is certainly a great endowment to our technical ability. But is it always an advantage? Certainly not,—only in the individual case; for general narcosis does not as a rule present so great dangers and ill consequences. This is the testimony of a great number of operators, native and foreign. Narcosis is more certain, for spinal anesthesia sometimes fails completely, either from defective technic or because the desired analgesia does not follow. It is a part of weakness for the operator to resort to narcosis in cases where he has promised the patient that none would be used. Another factor which speaks none too well for medullary anesthesia, may be the neglect of disinfection of the patient,—a view especially expressed by Professor Amann. It is clear that the operator may cut short aseptic procedures, by reason of the relatively brief duration of the medullary anesthesia. Kocher also expresses scruples of this character in his textbook of Operative Surgery, published three years ago, in which he states that perfect anesthesia may be followed by a strikingly unfavorable course of healing. We do not know whether he still holds to this view, but as far as known he uses medullary anesthesia little or not at all. It may not be a matter of defective asepsis at all, however, but of disturbances in local nutrition caused by the injection. Another point not to the credit of medullary anesthesia is this: the operator cannot proceed calmly with his intervention as long as he must hasten and hurry in order to complete his operation, before the anesthetic effect has worn off. It is also to be borne in mind that the psyche of the patient is essentially altered. Fully conscious, patients witness all the preparations of the operation, they hear all the directions given by the operator, they feel themselves strapped to the table,—circumstances which tend to give medullary anesthesia a cruel appearance.

How does this method stand in laparotomies,—operations which must so frequently be performed? Anesthesia is here successful as a rule and a moderate Trendelenburg posture is well borne. The pressure which forces intestinal loops into the operative field is extremely rare. One only was the insertion of the compress for covering the intestines rendered very difficult by the pressing of the patient. But a very troublesome disturbance sets in if a patient begins to retch and vomit, which happens often enough. Many vomit after general narcosis, but probably quite as many vomit sooner or later after medullary anesthesia.

We who practise gynecology, are in a high degree guided by results of palpation, and it seems very important to examine the patient before intervention, with the abdominal musculature relaxed. How interesting is it, then, to see our diagnosis straightway confirmed by operation! But with spinal analgesia the musculature does not notably relax, as I have convinced myself. I had opportunity once, indeed, to see a tumor, after spinal injection, become prominent to the view, which could not be seen before injection. This phenomenon, however, may have been due more to the position of the patient. Complete relaxation, by its absence, is a notable disadvantage for the gynecologist.

I am far from condemning spinal anesthesia, which in my opinion should be reserved for cases, in which general narcosis appears too risky. Again, the patient should be allowed to decide as to how she shall be anesthetised, for many have a dread of narcosis. But narcosis must be a necessity for all nervous and hysterical individuals. The old and weak tolerate spinal anesthesia well, and should be given the benefit of it. The combination of spinal anesthesia with morphin-scopolamin injections gave good results in two cases, but we know too little as yet for a judgment. Scopolamin, however, is not free from danger.

We have never as yet employed novocain in obstetrics, for puncture is rendered very difficult because of the abdominal and spinal anomalies of position in the lying-in woman. We may at least say that in gynecology novocain appears preferable to any other anesthetic.

